# *Tetrahymena* Comparative Genomics Database (TCGD): a community resource for *Tetrahymena*

**DOI:** 10.1093/database/baz029

**Published:** 2019-02-27

**Authors:** Wentao Yang, Chuanqi Jiang, Ying Zhu, Kai Chen, Guangying Wang, Dongxia Yuan, Wei Miao, Jie Xiong

**Affiliations:** 1 Key Laboratory of Aquatic Biodiversity and Conservation, Institute of Hydrobiology, Chinese Academy of Sciences, Wuhan, China; 2 University of Chinese Academy of Sciences, Beijing, China; 3 State Key Laboratory of Freshwater Ecology and Biotechnology, Wuhan, China; 4 CAS Center for Excellence in Animal Evolution and Genetics, Kunming, China; 5 Nextomics Biosciences Institute, Wuhan, China

## Abstract

Ciliates are a large and diverse group of unicellular organisms characterized by having the following two distinct type of nuclei within a single cell: micronucleus (MIC) and macronucleus (MAC). Although the genomes of several ciliates in different groups have been sequenced, comparative genomics data for multiple species within a ciliate genus are not yet available. Here we collected the genome information and comparative genomics analysis results for 10 species in the *Tetrahymena* genus, including the previously sequenced model organism *Tetrahymena thermophila* and 9 newly sequenced species, and constructed a genus-level comparative analysis platform, the *Tetrahymena* Comparative Genomics Database (TCGD). Genome sequences, transcriptomic data, gene models, functional annotation, ortholog groups and synteny maps were built into this database and a user-friendly interface was developed for searching, visualizing and analyzing these data. In summary, the TCGD (http://ciliate.ihb.ac.cn) will be an important and useful resource for the ciliate research community.

## Introduction

Ciliates are a large and diverse group of unicellular organisms (1). Within the cytoplasm of a single cell, ciliates have the following two types of differentiated nuclei that are structurally and functionally distinct: micronucleus (MIC) and macronucleus (MAC) (2). The MAC has exclusively somatic functions and directs gene expression (3). So far, draft MAC genomes of several ciliates have been sequenced in different groups (e.g. class) of ciliates, such as the *Tetrahymena thermophila* and *Paramecium tetraurelia* in Oligohymenophorea, *Euplotes octocarinatus*, *Stylonychia lemnae* and *Oxytricha trifallax* in Spirotrichea, *Stentor coeruleus* in Heterotrichida (4–9). Individual genome databases have been established for the ciliate community, such as the TGD Wiki (http://ciliate.org), ParameciumDB (http://paramecium.i2bc.paris-saclay.fr/) and EOGD (http://ciliates.ihb.ac.cn/database/home/#eo; 10–12). However, there is still a lack of comparative genomics database for closely related species within a ciliate genus similar to DroSpeGe (http://arthropods.eugenes.org/species/; 13) or PlasmoDB (http://plasmodb.org/plasmo/; 14).

The genus *Tetrahymena* contains more than 40 named species (15). Some species in this genus have a long and glorious history as model organisms, such as *T. thermophila*, a well-studied eukaryotic model organism (16), and *Tetrahymena pyriformis*, the most commonly used ciliated model for toxicology research (17). In 2006, the *T. thermophila* MAC genome was sequenced (4), and its corresponding database was established accessing to the community (TGD Wiki; http://ciliate.org). We subsequently constructed a functional genomics database TetraFGD (http://tfgd.ihb.ac.cn/; 18). Initialized in 2010, we sequenced and performed a comparative genomics analysis for 10 species in *Tetrahymena*, including the previous sequenced model organism *T. thermophila*, and 9 newly sequenced species. These 10 species are distributed among different subgroups and species complexes within the *Tetrahymena* genus. Among the nine newly sequenced species, three of them (*Tetrahymena malaccensis, Tetrahymena borealis* and *Tetrahymena elliotti*) were sequenced by the Broad Institute, and the rest six species (*T. pyriformis, Tetrahymena vorax, Tetrahymena canadensis, Tetrahymena empidokyrea, Tetrahymena shanghaiensis* and *Tetrahymena paravorax*) were sequenced by the Institute of Hydrobiology, Chinese Academy of Sciences. To facilitate the usage of these genome data, there is a need to build a comparative genomic database.

Here, we present the *Tetrahymena* Comparative Genomics Database (TCGD), which integrates genomic sequences, transcriptomic data, gene annotations, orthologs and synteny maps of 10 closely related *Tetrahymena* species. A user-friendly web interface has been implemented for data searching and visualization. We believe that this is an important online resource for the ciliate community.

## Materials and methods

### Data collection

The TCGD contains the following five main types of data: (i) sequence data, which include genome assemblies, predicted coding sequences (CDS) and protein sequences. Four MAC genomes including *T. borealis, T. elliotti, T. malaccensis* and *T. thermophila* were sequenced by the Broad Institute and can be accessed through the TGD Wiki (http://ciliate.org/index.php/home/downloads; 19). The other six genomes, sequenced by the Institute of Hydrobiology, Chinese Academy of Sciences, can be accessed through this database. The MAC genome sizes of 10 *Tetrahymena* species range from 84.9 Mb (*T. empidokyrea*) to 116.1 Mb (*T. pyriformis*; Table 1). In general, more than 200 000 protein-coding genes were predicted in all these 10 species (Table 1); (ii) gene annotations, which include homology-based gene annotations (e.g. best BLAST hit), protein domains, gene ontology (GO) and KEGG ortholog information. Protein domains were annotated using InterProScan (Version 5.19-58.0), which integrates information from ProDom, PANTHER, PROSITE, Pfam and SMART (20). In addition, *in silico* annotation on coiled-coil, signal peptides and transmembrane helices were also included in the database; (iii) synteny maps, which were detected using the MCScanX toolkit (21), based on ortholog groups generated by OrthoMCL (Version 2.0.9); (iv) transcriptomic data, which include the RNA-seq data at growth and starvation stages for each species; and (v) morphological data, including silver staining and scanning electron microscopy images.

**Table 1:** Basic statistics of MAC genomes of 10 *Tetrahymena* species

**Species**	**MAC genome size (Mb)**	**Gene number**
*T. thermophila*	103	26 996
*T. malaccensis*	106.7	24 866
*T. elliotti*	90.8	22 925
*T. pyriformis*	116.1	26 866
*T. vorax*	114.8	25 238
*T. borealis*	93.5	20 694
*T. canadensis*	103.4	25 188
*T. empidokyrea*	84.9	20 847
*T. shanghaiensis*	95.6	21 982
*T. paravorax*	108.4	25 551

### TCGD implementation

**Figure 1:**
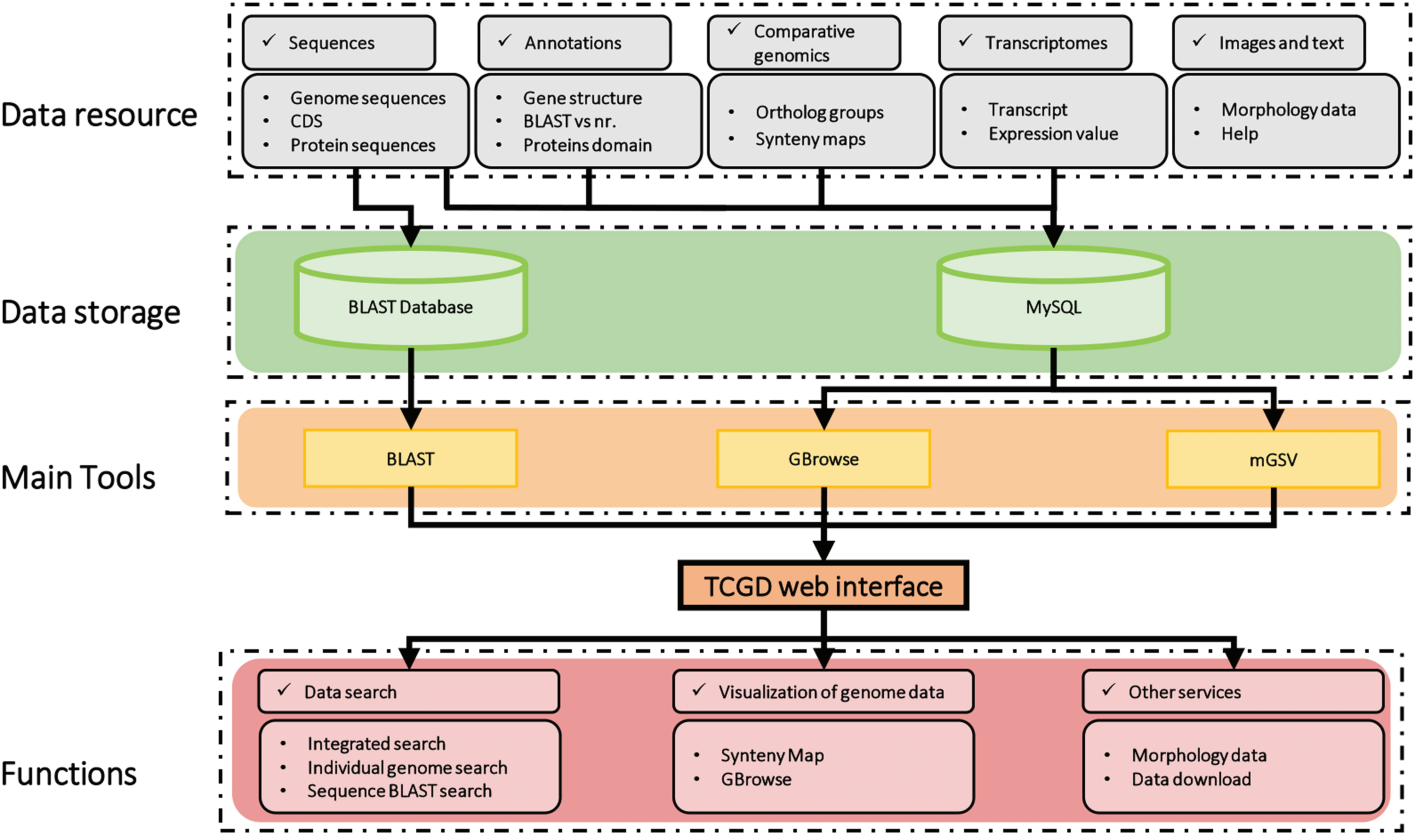
Schematic structure of the TCGD. A flow diagram shows the database architecture. Genome sequences, CDS and protein sequences were formatted as a BLAST database. Sequences, annotation information, comparative genomics data and transcriptomic data were stored in the MySQL database. GBrowse and mGSV were used for visualization of genome data and synteny map. Search and visualization allowed user to easily access the data resources in TCGD.

The schematic structure of TCGD is shown in Figure 1. The TCGD was built on the Linux operating system with Apache web server. All data are stored in a MySQL rational database management system. The TCGD web interface was developed with JavaScript/HTML to integrate all data resources for user-friendly searching and visualization. GBrowse software was used for visualization of genomic sequences, gene model and transcriptomic data (22). Multi-Genome Synteny Viewer (mGSV Version 2.1; 23), a web-based tool, was adapted to display the genomic features and their relative order in the genomes of the 10 *Tetrahymena* species. In addition, a standard NCBI BLAST server was set up to enable users to search for similar sequences and retrieve homologous genome components or regions in TCGD.

## Using the TCGD

The TCGD has a user-friendly web interface. Two main functions, including the search and visualization, have been implemented to facilitate data accessing.

### Data searching

**Figure 2:**
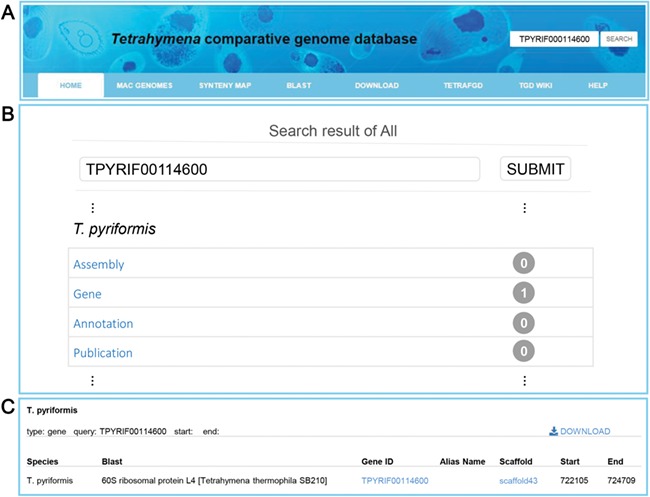
Search functions implemented into TCGD. (**A**) An integrated search box. (**B**) Screenshot of search result interface for gene TPYRIF00114600 through the integrated search box. (**C**) A brief gene description of TPYRIF00114600, including the species the gene belongs to, putative annotation based on NCBI BLAST hits and the gene location.

**Figure 3:**
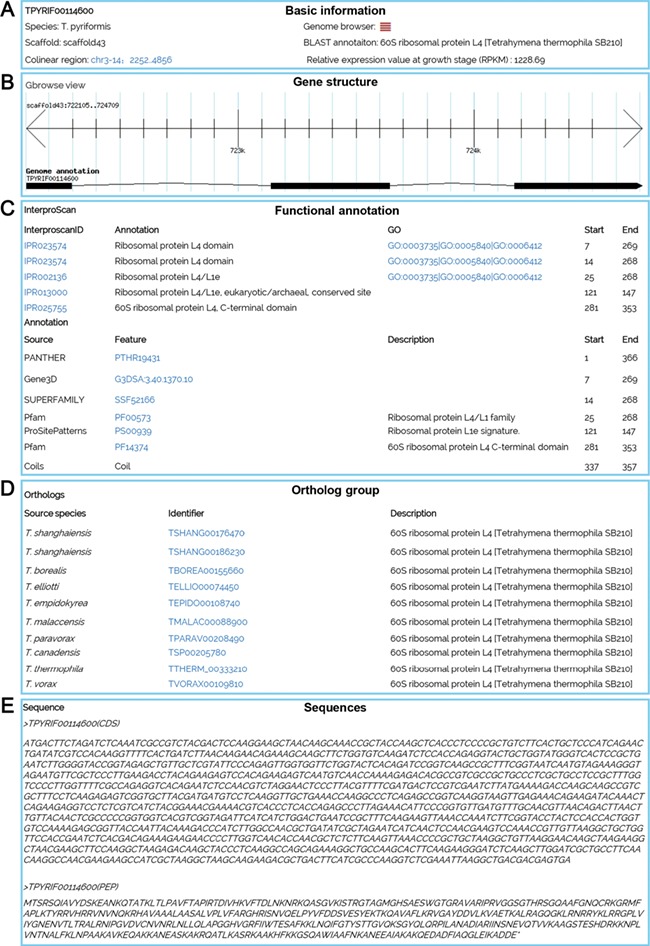
A gene details page for TPYRIF00114600, showing five types of data. (**A**) Basic information on the gene, such as the species, putative annotation based on NCBI BLAST hits, and the gene location. (**B**) A snapshot of the gene structure with a hyperlink to GBrowse. (**C**) Annotation with InterProScan for protein domains, GO and KEGG function. (**D**) Homolog information for all 10 species based on OrthoMCL ortholog groups. (**E**) The predicted CDS and protein sequences.

Multiple search functions have been implemented to enable researchers to obtain useful information (Figure 1). Firstly, an integrated search box (located in the top right corner of the home page) enables whole database searching through the categories ‘Gene ID,’ ‘Scaffold ID,’ or ‘key words’ (Figure 2A). Gene ID is recommended. The general naming rule for genes in nine species (except for *T. thermophila*) is a prefix indicating the species plus a suffix consisting of eight-digit numbers. The prefixes are TBOREA for *T. borealis*, TELLIO for *T. elliotti*, TEMPID for *T. empidokyrea*, TMALAC for *T. malaccensis*, TPARAV *for T. paravorax*, TPYRIF for *T. pyriformis*, TSHANG for *T. shanghaiensis*, TSP for *T. canadensis* and TVORAX for *T. vorax*. The suffixes indicate the order of genes in the assembled scaffolds. After searching with a specified Gene ID, the hit will be displayed (Figure 2B), with a hyperlink (Figure 2C) to a page containing detailed information of the gene. The following five types of information are presented on this page (Figure 3): (i) a brief description of the gene, including the species, putative annotation based on NCBI BLAST, its location, the assembled scaffold and a hyperlink with coordinates to the synteny map (Figure 3A); (ii) a snapshot with hyperlink shows the gene structure and could further forward the user to GBrowse (Figure 3B); (iii) protein domain, GO and KEGG functional annotations (Figure3C); (iv) homolog information for all 10 species based on OrthoMCL ortholog groups (Figure 3D); and (v) the predicted CDS and protein sequences (Figure 3E). In addition, a ‘MAC Genomes’ tab navigation bar in the home page directs the user to the MAC genome database for each species (Figure 1). The TCGD also allows BLAST search of sequence against either individual genomes or all 10 genomes**.**

### Visualization of genomic data

**Figure 4:**
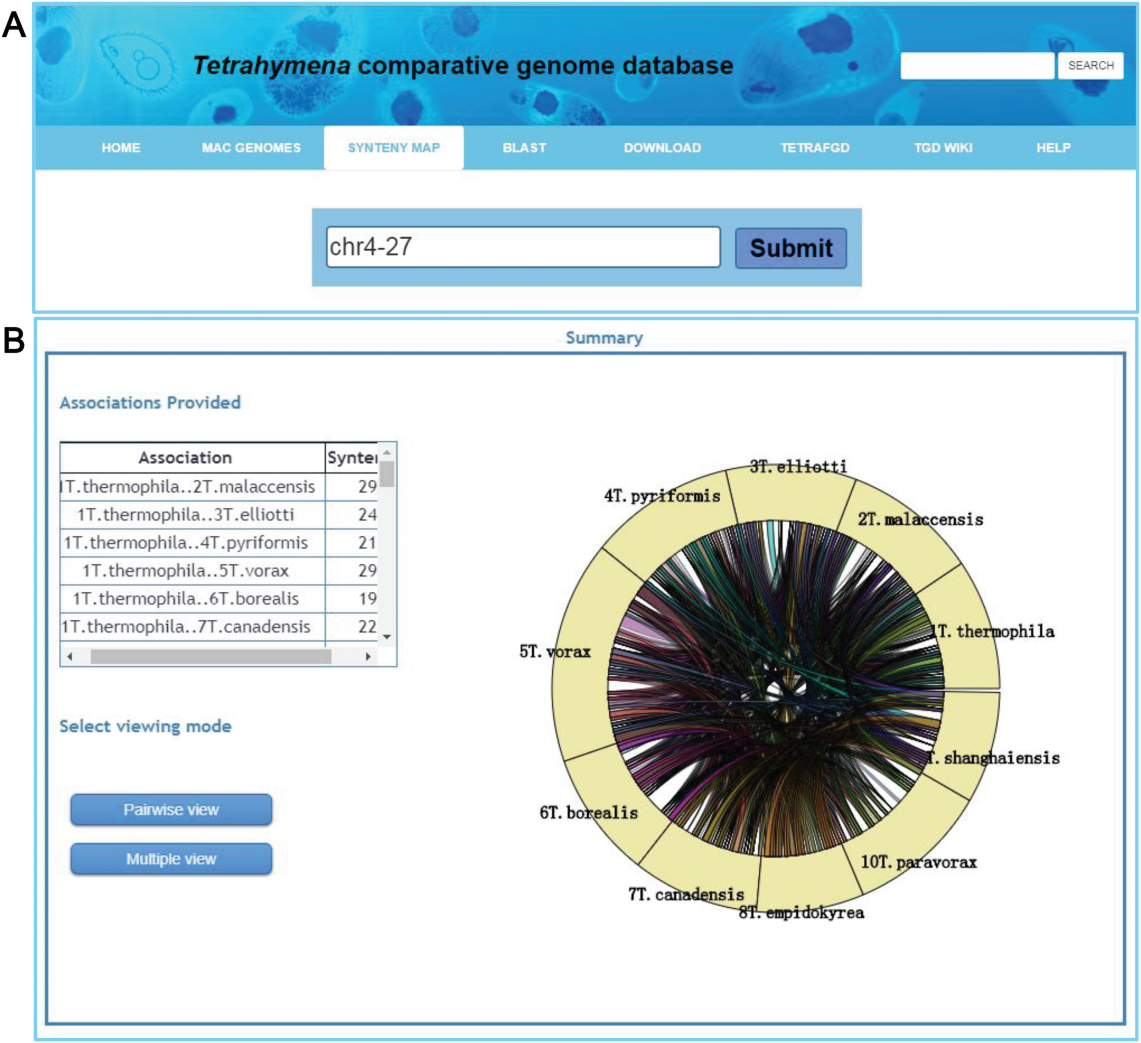
Visualization of a synteny map in TCGD. (A) The ‘collinear block ID’ or ‘Gene ID’ is inserted into the search box to acquire a synteny map for 10 *Tetrahymena* species. (**B**) A summary page shows genome associations and the number of genes for each genome pair for collinear block ID chr4-27. A circular diagram shows a general overview of the associations. To obtain the full synteny display, users can choose to enter either ‘Pairwise view’ or ‘Multiple view’ mode.

An important function for comparative genomics analysis in TCGD is visualization of the synteny maps between genomes of all 10 species. In TCGD, mGSV tool was implemented for browsing the synteny maps. For this, we divided genome-wide collinear relationships into blocks that were generated based on MAC chromosomes of the model species, *T. thermophila*. In *T. thermophila*, the newest assembly suggested that the MAC has 181 chromosomes (including the rDNA minichromosome). We first aligned all assembled scaffolds in other species onto the 181 MAC chromosomes of *T. thermophila* using LASTZ, and then ordered the assembled scaffolds according to the alignment location in *T. thermophila* MAC chromosomes. In some cases, only one homologous scaffold in other species has been found for two *T. thermophila* MAC chromosomes*,* meaning that a single chromosome in another species will be represented by two in *T. thermophila*. In these cases, we artificially merged the two *T. thermophila* chromosomes to form a block. A total of 173 blocks (in general, each one representing a MAC chromosome) were obtained and synteny maps were generated for incorporating them into mGSV. All 173 collinear blocks were named according to their order on the MIC chromosomes of the *T. thermophila* MIC. The principle for searching the collinear block is that a ‘chrX-Y’ style ID should be provided, in which ‘X’ represents the MIC chromosome number (five MIC chromosomes, numbered 1–5) and ‘Y’ represents the order on the MIC chromosome. A list of genes in each block was also provided. Besides the block ID, a Gene ID can also be used for searching the synteny map (Figure 4A). After selecting a block or searching by Gene ID, a circular layout containing all conserved genes in the block among 10 species is shown (Figure 4B), with two visualization modes available to browse the synteny maps ‘Pairwise view’ and ‘Multiple view’ (Figure 4B).

**Figure 5:**
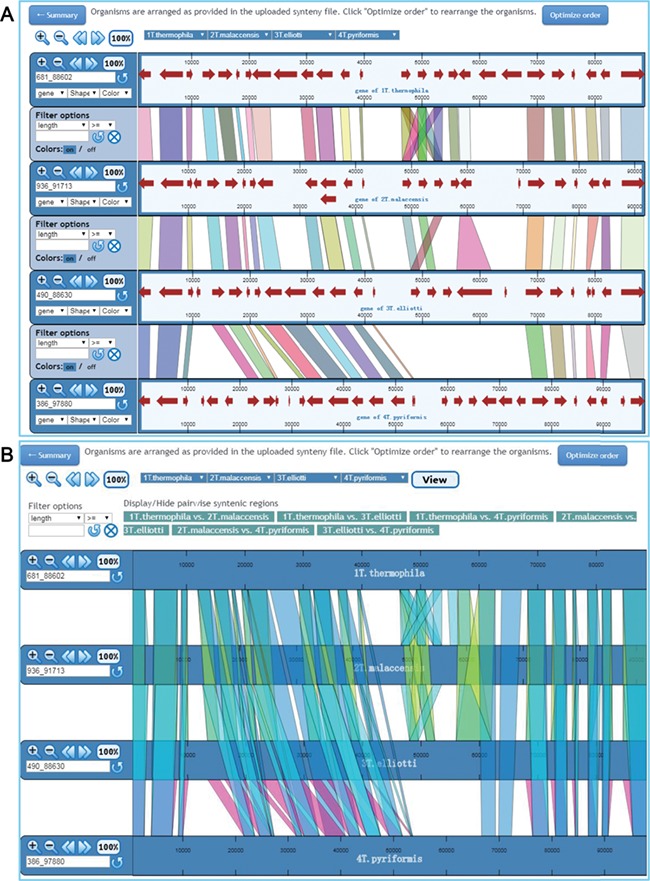
Visualization of synteny map in ‘Pairwise view’ mode and ‘Multiple view’ mode. (**A**) In ‘Pairwise view’ mode, genes are shown as lines between adjacent genomes. Genomes can be rearranged, removed or shown more than once. Genome control panels on the left side of the interface allow the genome viewing range to be adjusted. Master controls at the top apply to all genomes. By using the control panel on the left, users can choose the shape and color of genes. Regions of visible synteny can be filtered based on the numerical criteria specified for genes. (**B**) In ‘Multiple view’ mode, conserved genes across all selected genomes are shown. The regions associated with one or more specific genome pairs can be hidden using the buttons above the synteny display. Genomes can also be rearranged or removed, and each genome is displayed only once.

The ‘Pairwise view’ mode shows the synteny maps between adjacent genomes (Figure 5A). Multiple pull-down menus located at the top of the synteny browser enable users to choose specific genomes to display in any order they wish. These pull-down menus can be added or removed using the ‘Insert’ or ‘Delete’ option so that each genome can be shown more than once or removed if necessary. Buttons at the top left corner allow users to change the view for all genomes displayed by zooming in/out, moving left/right. The display for each genome consists of two parts: the control panel on the left and the synteny display on the right. The left control panel allows users to zoom in/out, move left/right or select specific regions for display. Users can also filter the conserved genes based on the length of conserved regions, as recorded in the synteny files. In the synteny display window, each genome is represented as a horizontal ruler with tick marks showing its genome position and with genes displayed as colored arrowed blocks. The shapes and colors of the gene display can be easily changed via the control panel. When the mouse pointer moves to a gene, the gene name appears. A detailed description of each gene is obtained by clicking on the gene. Each conserved gene is colored differently in the default setting, but users can change the displayed regions to a uniform color via the ‘Colors’ option. We have also provided the coordinates of each in the gene detail page; user can easily find the exact location of a gene in the synteny map using these coordinates.

In ‘Multiple view’ mode, all synteny maps are shown for each pair of genomes in all selected species (Figure 5B). Users can switch on/off the display of conserved genes between any pair of genomes. As in ‘Pairwise view’ mode, any genome can be added or removed from the display. A single filter panel at the top of the synteny view can be used to filter the conserved genes. By default, all genomes are shown in the order in which they are specified in the synteny files. In both viewing mode, an ‘Optimize order’ option is provided to rearrange the order based on an algorithm developed in mGSV.

In addition, GBrowse was built into the TCGD to visualize genomic data for individual species. Users can click the ‘MAC Genomes’ tab to select a species and then visualize the genome sequence through selecting the navigation tab ‘GENOME BROWSER’ on the homepage of each species. Three tracks [putative gene model, RNA-seq coverage plot and expressing value (RPKM)] are shown by default.

### Other services in the TCGD

The TCGD also provides morphological data in the form of silver staining and scanning electron microscopy images, which allow users to familiarize themselves with the morphology of these species.

## Discussion

*T. thermophila* is a well-studied model system in ciliates. After sequencing of the *T. thermophila* MAC genome in 2006, two important *Tetrahymena* databases have been established: the TGD Wiki, which contains genomic information; and TetraFGD, a functional genomics database that enables researchers to access gene expression, gene network and proteomics data. Sequencing of the MAC genomes of other nine species provided an opportunity to build the TCGD (similar to the *Drosophila* and *Plasmodium* databases). The TCGD aims to provide basic genomic information and comparative genomics analysis of 10 *Tetrahymena* species. Users can obtain comprehensive information on genes of interest through the TCGD. We believe that the TCGD represents an important database for the *Tetrahymena* and ciliate research community.

*Tetrahymena* have both a MIC and MAC in a single cell. Although the MAC genomes of multiple species have been sequenced, so far the MIC genome of only *T. thermophila* has been sequenced using the Illumina platform. As DNA sequencing technology develops, PacBio and Nanopore sequencing platforms could be used to obtain more complete MIC genome sequences of other *Tetrahymena* species. Future efforts of the TCGD will focus on incorporating the MIC genomes of more *Tetrahymena* species and on facilitating MIC and MAC comparative genomics analysis between different species.

## Availability of supporting data

The TCGD is freely accessible as a web application at http://ciliate.ihb.ac.cn. All data, including the genome sequences, CDS, protein sequences, functional annotation files and ortholog groups, are available for download, e.g. http://ciliate.ihb.ac.cn/tcgd/database/download/#th.

## Author contributions

W.M. and J.X. conceived the project. W.T.Y., Y.Z., K.C., G.Y.W. and D.X.Y. performed the data analysis and constructed the database. C.Q.J. collected the morphological pictures. W.M., J.X. and W.T.Y. wrote the paper.

## Notes

**Database URL: http://ciliate.ihb.ac.cn**
